# c-Fos expression following context conditioning and deep brain stimulation in the bed nucleus of the stria terminalis in rats

**DOI:** 10.1038/s41598-020-77603-z

**Published:** 2020-11-25

**Authors:** Kelly Luyck, Isabelle Scheyltjens, Bart Nuttin, Lutgarde Arckens, Laura Luyten

**Affiliations:** 1 Experimental Neurosurgery and Neuroanatomy, KU Leuven, UZ Herestraat 49, PB 7003, 3000 Leuven, Belgium; 2 Animal Physiology and Neurobiology, KU Leuven, Naamsestraat, PB 2467, 3000 Leuven, Belgium; 3 Leuven Brain Institute, Herestraat 49, PB 1021, 3000 Leuven, Belgium; 4 Centre for Psychology of Learning and Experimental Psychopathology, KU Leuven, Tiensestraat 102, PB 3712, 3000 Leuven, Belgium; 5 VIB Center for Inflammation Research, Vrije Universiteit Brussel, Myeloid Cell Immunology, Pleinlaan 2, 1050 Brussel, Belgium

**Keywords:** Translational research, Neurology, Anxiety, Neuroscience, Fear conditioning

## Abstract

Deep brain stimulation (DBS) in the bed nucleus of the stria terminalis (BST), a region implicated in the expression of anxiety, shows promise in psychiatric patients, but its effects throughout the limbic system are largely unknown. In male Wistar rats, we first evaluated the neural signature of contextual fear (*N* = 16) and next, of the anxiolytic effects of high-frequency electrical stimulation in the BST (*N* = 31), by means of c-Fos protein expression. In non-operated animals, we found that the left medial anterior BST displayed increased c-Fos expression in anxious (i.e., context-conditioned) versus control subjects. Moreover, control rats showed asymmetric expression in the basolateral amygdala (BLA) (i.e., higher intensities in the right hemisphere), which was absent in anxious animals. The predominant finding in rats receiving bilateral BST stimulation was a striking increase in c-Fos expression throughout much of the left hemisphere, which was not confined to the predefined regions of interest. To conclude, we found evidence for lateralized c-Fos expression during the expression of contextual fear and anxiolytic high-frequency electrical stimulation of the BST, particularly in the medial anterior BST and BLA. In addition, we observed an extensive and unexpected left-sided c-Fos spread following bilateral stimulation in the BST.

## Introduction

The bed nucleus of the stria terminalis (BST) has emerged as one of the key players in anxiety and stress responses in rodent, nonhuman primate and human studies^1–12^. Although there is an ongoing debate about the prerequisites of BST recruitment^6,13^, exposure to a fear-conditioned context is generally accepted to elicit sustained anxiety-like behavior involving the BST^7,14,15^. Note that we will refer to context-conditioned rats as being anxious, understood as showing fearful responses to a potentially dangerous context, characterized by agitation and apprehension with high temporal uncertainty of when and whether an aversive event might occur^7,16–18^. We recently showed that bilateral high-frequency electrical stimulation of the BST reduces the expression of contextual fear in such a conditioning procedure^19^. These findings are in line with the clinical benefits of deep brain stimulation (DBS) of the BST in patients suffering from obsessive–compulsive disorder (OCD), showing clear anxiolytic effects during stimulation^20^. Nevertheless, it is important to note that the mechanisms of high-frequency electrical stimulation are not yet fully understood. Studies of the cellular and molecular physiology of DBS suggest that it may mimic the effect of a lesion, by inactivating the area immediately surrounding the electrode contact (e.g., through a depolarization block or local release of GABA)^21,22^. Whether these are the mechanisms that account for the anxiolytic effects of DBS in the BST is currently an unanswered question, but it seems possible that DBS may ease an overactive BST. In previous studies, we found that the BST was indeed hypermetabolic in anxious rats and that electrolytic lesions in the BST had anxiolytic effects^14,15^. Despite the clinical promise of BST stimulation in both rodent and patient studies, its effects on brain-wide projections are largely unknown. Given that the BST is a well-connected brain region that does not function in isolation^12,23^, it seems worthwhile to investigate whether some of these connections play a role in the observed anxiolytic effects. To the best of our knowledge, such effects of electrical BST stimulation in anxious rodents have never been reported, and only one positron-emission tomography (PET) study evaluated this in psychiatric patients^24^. Procedural disadvantages of this study were that not all patients were stimulated in the BST (about half of them received DBS in the anterior limb of the internal capsule), and that images were acquired in a ‘neutral’ state (no disorder-specific triggers were presented). The main findings were stimulation-induced metabolic decreases in the anterior cingulate (which was increased pre-operatively) and in the prefrontal and orbitofrontal cortices.

In contrast to this limited knowledge on the neural effects of BST stimulation, a vast amount of research has been dedicated to physiological BST functioning using various anxiety and stress paradigms. The majority of these studies report on the bilateral BST as a whole, without differentiating between both hemispheres. Recent evidence, however, suggests that structural and functional asymmetries in the brain go beyond those commonly described for movement and visual systems^25,26^. Indeed, visual inspection of imaging data from context conditioning or threat monitoring studies suggests that BST activity was more prominent in the left compared to the right hemisphere^7,14,27,28^. Moreover, a recent study in healthy participants revealed that structural connectivity between the BST and its projections is not identical in both hemispheres^8^. For example, the left nucleus accumbens (NAc), left amygdala and left caudate showed stronger connectivity with the BST compared to their right counterparts. Except for this study, a potential asymmetry in BST activity has been largely neglected in prior research, while differences between the left and right basolateral amygdala (BLA) have received more attention, typically indicating a greater involvement of the right hemisphere in rodents and humans^10,29–34^. Combined, these data indicate some asymmetry in the BST and BLA, and suggest that bilateral manipulations of the BST might produce partly lateralized effects on its connections.

In the present study, we evaluated the neural signature of contextual fear and of the anxiolytic effects of electrical stimulation in the BST. To this end, we assessed the expression of the early gene c-Fos, which is induced in neurons by a wide variety of external stimuli^35–37^ and is a high-resolution marker of neural activity^38–42^. c-Fos protein expression was measured in several regions of interest: the BST, BLA, NAc and infra- and prelimbic cortex. The BLA is an obvious region of interest in view of its central role in the fear and anxiety circuitry, mediating both fear learning and its expression^2,43,44^. The contribution of the NAc to conditioned responding is currently less well-understood, although it has been implicated in the expression of contextual fear as well as in avoidance behavior, an important aspect of (clinical) anxiety^45–47^. The NAc is moreover of interest because it has been proposed as a target for DBS in psychiatric patients^48,49^, comparable to our clinical studies with DBS in the BST^20^. The infra- and prelimbic cortices are generally considered to be part of the rodent prefrontal cortex and are therefore an interesting area to evaluate, also in light of the abovementioned PET study in patients treated with DBS^24^. Moreover, these regions have emerged as important players in the reduction and promotion of fear memory expression, respectively^43^. In a first experiment, with intact animals that did not undergo surgery, we quantified c-Fos expression in rats expressing contextual fear versus non-anxious controls, allowing for an evaluation of neuronal activity in an intact BST. In a second experiment, animals were implanted with bilateral electrodes in the BST, underwent contextual fear conditioning and received anxiolytic, bilateral BST or sham stimulation throughout the final test session^19^. c-Fos expression during this session was analyzed to shed light on the neural signature of the observed anxiolytic effects. In both experiments, c-Fos outcome was segregated between left and right hemispheres, to allow for a detailed analysis of possible asymmetric profiles in neuronal activation.

## Materials and methods

### Subjects

Forty-seven male Wistar rats (± 250–270 g at the start of the experiments) were used. This project was approved by the KU Leuven animal ethics committee and was in accordance with the Belgian and European laws, guidelines and policies for animal experimentation, housing and care (Belgian Royal Decree of 29 May 2013 and European Directive 2010/63/EU on the protection of animals used for scientific purposes of 20 October 2010).

### Surgery

Animals in Experiment 2 were implanted with bilateral electrodes in the anterior-medial division of the BST (STMA), as described before^19^. We used custom-made Pt/Ir electrodes with a diameter of 127 µm (AM Systems, Sequim, WA, USA), and coordinates were 0.0 mm (anterior–posterior), 3.4 mm (medio-lateral) and 6.3 mm (subdural), under a 20° angle^50^. Animals were allowed to recover for 6–7 days before the start of behavioral experiments.

### Context conditioning

We used a context conditioning protocol that has been described previously^18,51,52^ (see Supplement). Briefly, animals were conditioned and tested in a startle box, i.e., a sound-attenuating chamber containing a small animal cage, in which foot shocks and startle probes could be delivered, and two behavioral measurements (startle reflex amplitude and freezing behavior) were recorded. In Experiment 1, rats underwent a Habituation session (20 min) on day 1, during which they were placed in the startle box, and after 5 min of acclimation, presented with 30 startle stimuli (100 dB, 50 ms). On day 2, during the Pre-test (identical to Habituation), baseline freezing (during 5-min acclimation) and startle responses were collected. Training (30-min session) on day 3 consisted of 10 foot shocks (0.8 mA, 250 ms) for ANX rats (n = 9) or no shocks for CTRL animals (n = 7). During the Post-test on day 4 (identical to Pre-test), ANX animals are expected to express anxiety in the context where they previously received electrical shocks, as quantified by increased freezing during acclimation and startle potentiation.

In Experiment 2, all rats [STIM (n = 17) and SHAM (n = 14)] received shocks during Training and were therefore conditioned to the context. STIM animals received electrical BST stimulation prior to and during the Post-test, whereas SHAM rats did not.

### Electrical stimulation

In Experiment 2, the implanted electrodes were connected to a stimulator (STG 4008, MultiChannel Systems MCS GmbH, Reutlingen, Germany) through a wired 6-channel system (363-SL/6, Plastics1, Roanoke, VA, USA), on each day of the behavioral protocol. All animals were connected starting one hour prior to each behavioral test session in a cage similar to their home cage, until completion of the session in the startle box. Actual stimulation in STIM animals only took place on the Post-test day (see Supplement). To evaluate and allow for attenuation of potential side effects, stimulation was initiated in a home cage 1 h before the start of the Post-test, as described previously^19^.

### Immunohistochemistry

**Figure 1:**
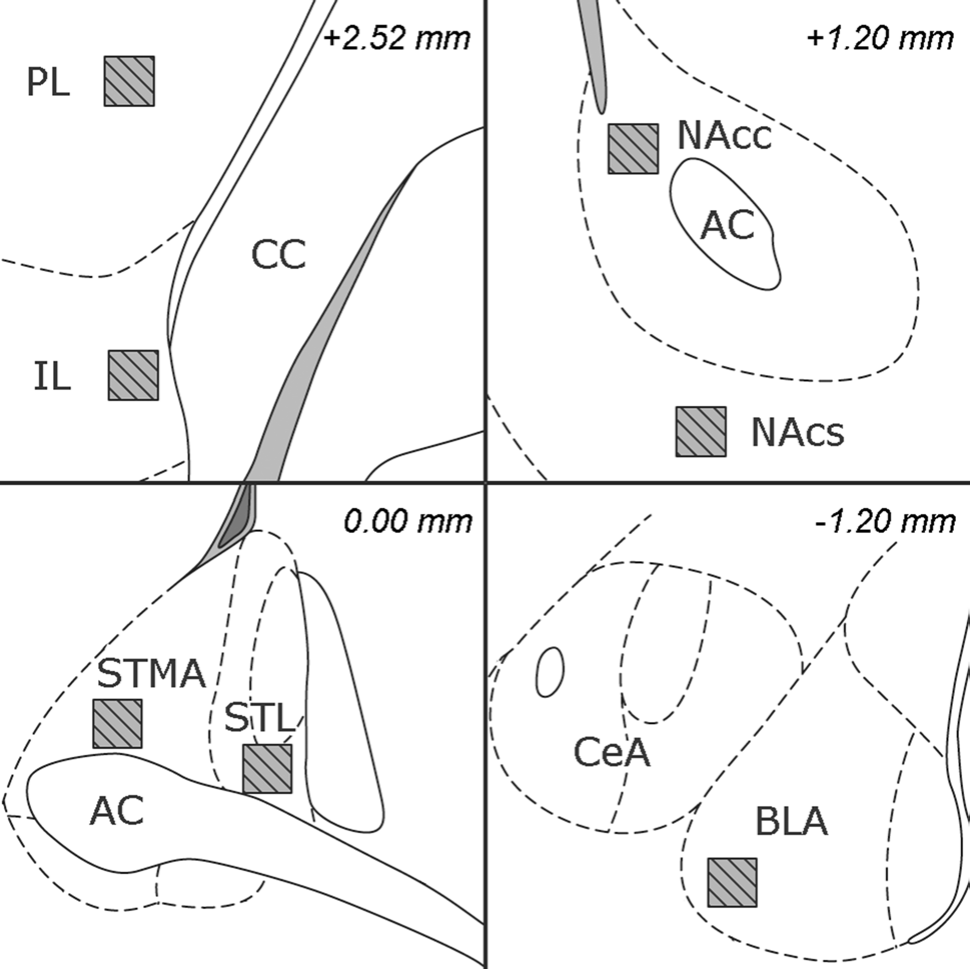
Regions of interest (ROIs) (200 × 200 µm) for immunohistochemistry. ROIs are indicated with hatched squares. Distance of the cross-section to bregma in the atlas is shown in the right upper corners. AC: anterior commissure, BLA: basolateral amygdala, CC: corpus callosum, CeA: central amygdala, IL: infralimbic cortex, NAcc: core of the nucleus accumbens, NAcs: shell of the nucleus accumbens, PL: prelimbic cortex, STL: lateral division of the bed nucleus of the stria terminalis, STMA: medial division of the anterior bed nucleus of the stria terminalis. Adapted from Paxinos and Watson^50^.

In Experiment 1, six representative rats of both groups were selected to reflect the group average for freezing and startle measurements as accurately as possible (ANX: n = 6, CTRL: n = 6). Note that vibratome sectioning and subsequent c-Fos analysis was only conducted for this subset. For Experiment 2, to increase power, all animals with correct electrode placement were included (STIM: n = 14, SHAM: n = 11). Two hours after initiation of the Post-test, animals were sacrificed, brains were dissected and processed for immunohistochemical analysis (see Supplement). Briefly, coronal sections were stained for c-Fos^53^, NeuN and GFAP. In Experiment 1, we evaluated NAc (core and shell), BLA and BST (STMA and lateral anterior division (STL)) activity. In Experiment 2, analysis of the BST was hampered by electrode placement, thus we evaluated the BLA, NAc, infralimbic and prelimbic cortices (IL, PL). Confocal images were obtained in the abovementioned regions of interest (ROIs) (Fig. 1) and neuronal c-Fos expression was assessed. For each structure, all slices were stained simultaneously and imaged at fixed intensity settings, allowing for direct comparison of c-Fos intensities between groups and hemispheres within one brain region, thereby providing information about the degree of c-Fos expression, rather than the number of positive cells.

### Statistical analysis

Percentage freezing during acclimation and startle reflex amplitudes were expressed and analyzed as difference scores between Post- and Pre-test^14,19^. Unpaired two-tailed t-tests were implemented to compare these behavioral measurements, as well as c-Fos intensities between ANX and CTRL (Experiment 1) or STIM and SHAM groups (Experiment 2). Grubbs’ outlier tests were used to exclude animals with significantly deviant c-Fos values. A 2-way repeated measures ANOVA (RM-ANOVA) with factors ‘Hemisphere’ and ‘Group’ was conducted to evaluate c-Fos expression within and between hemispheres, followed up with Sidak post-hoc tests. GraphPad Prism (version 8.02, GraphPad Software) was used for statistical analyses and to create graphs. Effect sizes (Cohen’s *d*) were calculated according to Lakens^54^. Significance levels were set at p < 0.05.

## Results

### Experiment 1

#### Behavior

Baseline startle values (during Pre-test) did not differ between both groups (CTRL: 273 ± 122, ANX: 243 ± 104, mean ± SD; *t*_(14)_ = 0.54, p = 0.60) and baseline freezing was low in all animals (1% ± 2%).

**Figure 2:**
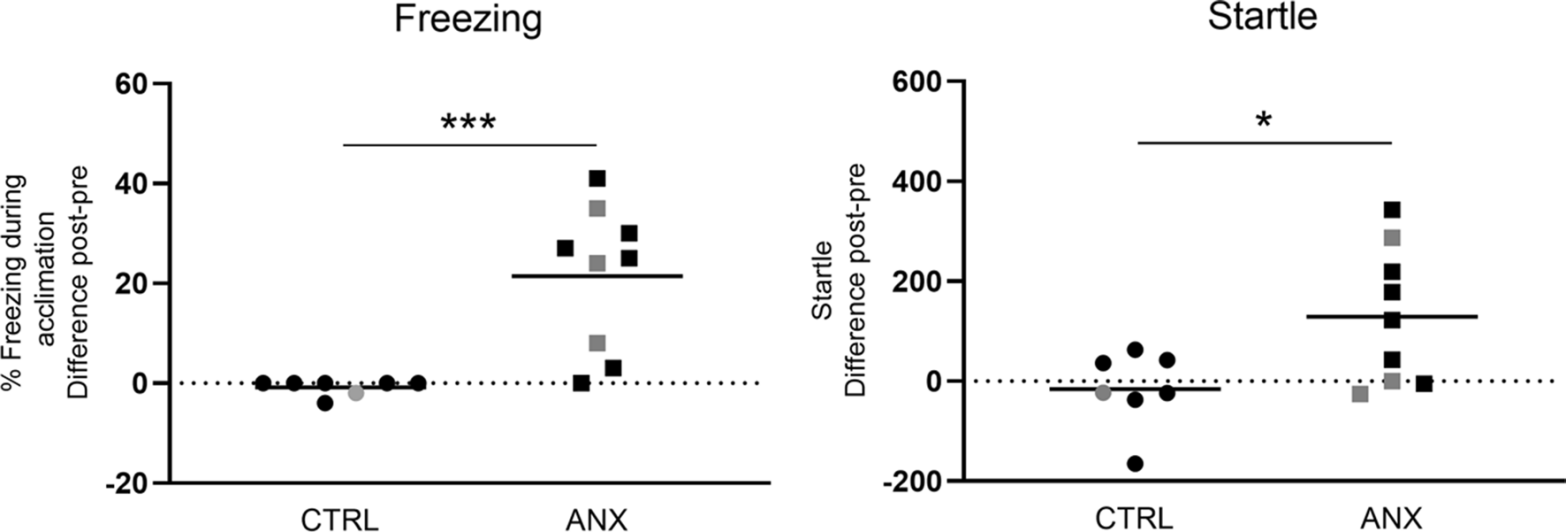
Percentage freezing during acclimation (left panel) and startle response (right panel) in Experiment 1. Difference scores of Post- minus Pre-test are shown as individual data points and means for CTRL (n = 7) and ANX (n = 9) animals. Rats indicated with black symbols were included for further c-Fos analysis (CTRL, n = 6; ANX, n = 6), *p < 0.05; ***p = 0.001.

At Post-test, both freezing and startle responses were significantly higher in ANX than CTRL animals (*t*_(14)_ = 4.03, p = 0.001 and *t*_(14)_ = 2.51, p = 0.025, respectively), indicating successful expression of contextual fear in ANX rats (Fig. 2). Next, we selected a subset of six animals per group for immunohistochemical analyses (Fig. 2, black data points). Also in these smaller groups, freezing (*t*_(10)_ = 3.28, p = 0.008) and startle (*t*_(10)_ = 2.66, p = 0.02) were significantly higher in ANX than CTRL animals.

#### Immunohistochemistry

All regions of interest are discussed here, but bar plots are only shown for ROIs with significant group differences. See Supplement for a detailed overview of all analyzed regions (Suppl. Table 1 and Suppl. Fig. 1).

**Figure 3:**
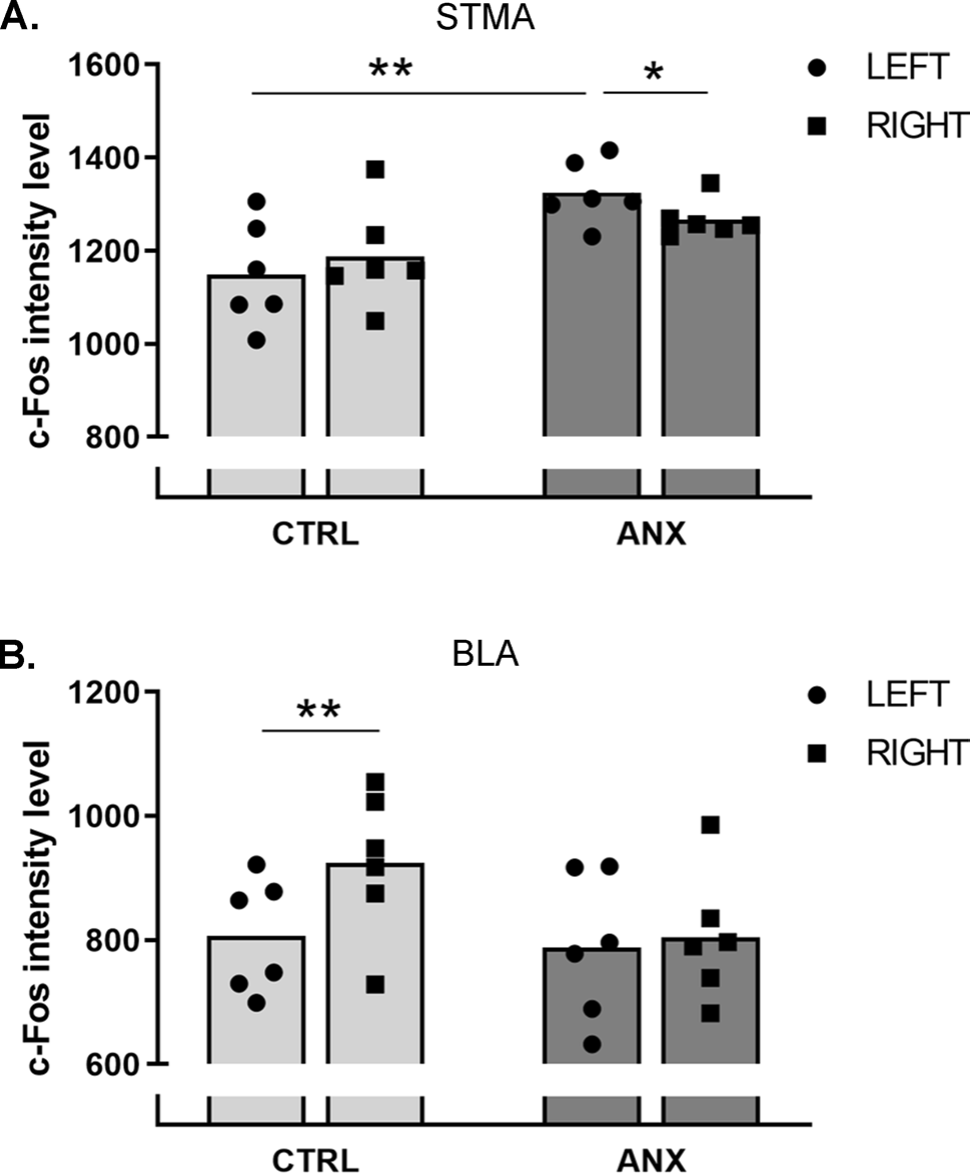
C-Fos intensities in the STMA (**A**) and BLA (**B**) in Experiment 1. Data are shown as individual data points (average of three consecutive slices) and means for CTRL (n = 6) and SHAM (n = 6) animals. BLA: basolateral amygdala, STMA: medial division of the anterior bed nucleus of the stria terminalis, *p < 0.05, **p < 0.01.

In the bed nucleus of the stria terminalis, medial anterior division (STMA), we found a significant main effect of ‘Group’ (*F*_(1,10)_ = 6.96; p = 0.03) and a significant interaction between ‘Group’ and ‘Hemisphere’ (*F*_(1,10)_ = 13.58; p = 0.004). Post-hoc analysis revealed differences between left and right within the ANX group (p = 0.02). In addition, c-Fos expression was significantly increased in the left hemisphere of ANX animals, compared to CTRL (p = 0.004) (Fig. 3A). In the lateral division of the BST (STL), we did not observe any significant differences between groups or hemispheres (Suppl. Table 1 and Suppl. Fig. 1).

In the basolateral amygdala (BLA), we found a main effect of ‘Hemisphere’ (*F*_(1,10)_ = 13,66; p = 0.004), but not of ‘Group’. In addition, we found a significant interaction between both factors (*F*_(1,10)_ = 7.82; p = 0.02). Post-hoc analysis revealed stronger c-Fos expression in the right than left hemisphere of CTRL animals (p = 0.002) (Fig. 3B).

In the NAc (core and shell), we did not observe any differences in c-Fos expression between hemispheres or groups (Suppl. Table 1). We did find a significant main effect of ‘Hemisphere’ in the shell region (*F*_(1,10)_ = 6,77; p = 0.03), with the left hemisphere displaying stronger c-Fos expression in both groups (Suppl. Fig. 1).

### Experiment 2

**Figure 4:**
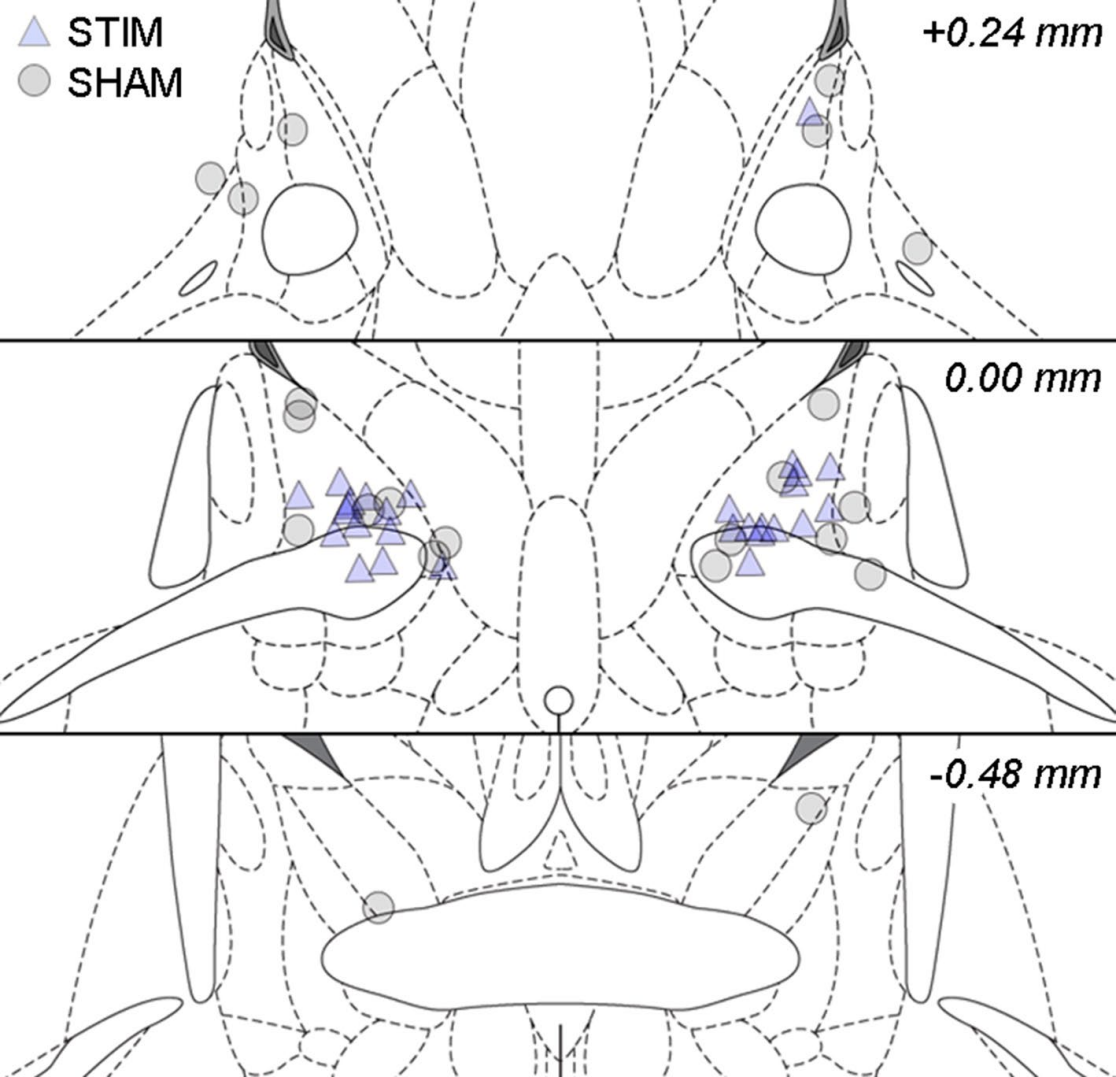
Reconstruction of electrode tip locations in Experiment 2. Triangles represent STIM animals (n = 14), circles represent SHAM animals (n = 11). Coronal slices shown from top to bottom are + 0.24 mm, 0.00 mm and − 0.48 mm, with respect to bregma. Figure adapted from Paxinos and Watson^50^.

Five animals (3 STIM, 2 SHAM) were excluded from further analyses due to incorrect electrode placement. In two animals, electrodes were positioned too anteriorly, comprising the NAc area, while other electrodes were placed either too ventrally or dorsally. In addition, one SHAM animal was excluded based on outlying Pre-test startle values (> mean + 2SD). In total, we included 14 animals in the STIM group, and 11 animals in the SHAM group (Fig. 4).

#### Behavior

Baseline startle values (during Pre-test) did not differ between both groups (SHAM: 220 ± 128, STIM: 264 ± 127, mean ± SD; *t*_(23)_ = 0.87, p = 0.40) and baseline freezing was low in all animals (5% ± 10%).

**Figure 5:**
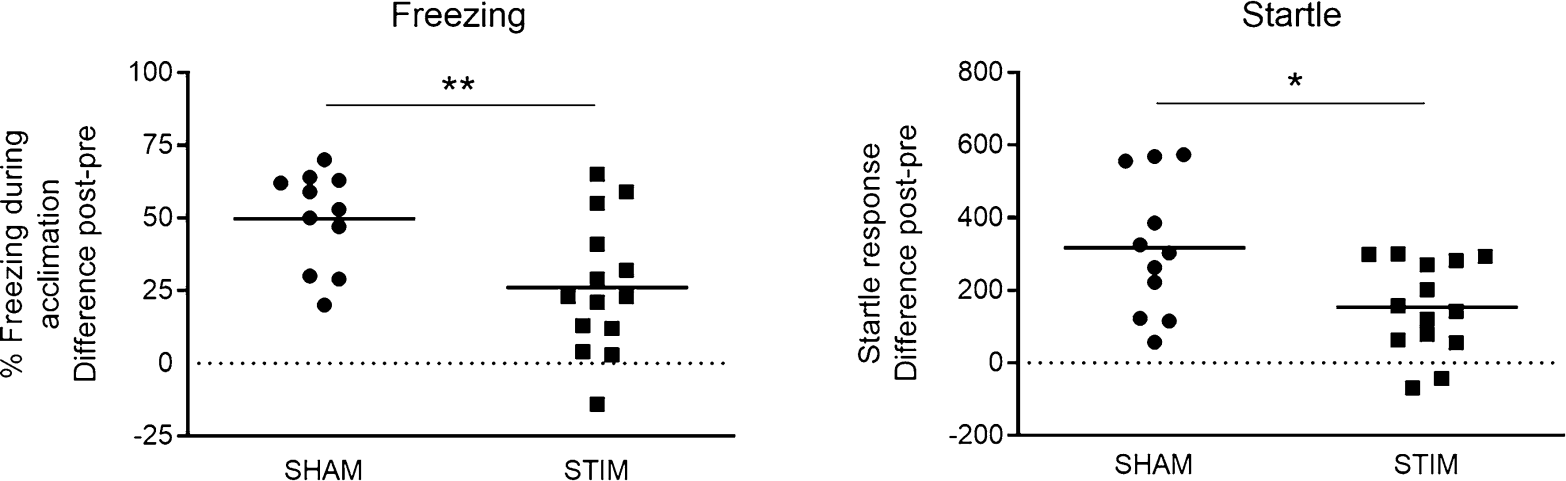
Percentage freezing during acclimation (left panel) and startle response (right panel) in Experiment 2. Difference scores of Post- minus Pre-test are shown as individual data points and means for SHAM (n = 11) and STIM (n = 14) animals, *p < 0.05; **p < 0.01.

At Post-test, STIM animals showed reduced freezing (*t*_(23)_ = 2.9; p = 0.008) and startle responses (*t*_(23)_ = 2.6; p = 0.02) compared with SHAM rats (Fig. 5), indicative of anxiolytic effects of high-frequency electrical stimulation in the BST.

#### Immunohistochemistry in regions of interest

All ROIs are discussed below and corresponding c-Fos intensities are listed in Table 1, together with the significance of between-group differences. See Supplement for bar plots showing individual data points (Suppl. Fig. 2).

**Table 1:** Overview of neuronal c-Fos intensities in Experiment 2.

Experiment 2	SHAM	STIM	Group significance
**BLA**			
Left	**1594** ± 160	**2195** ± 317	p < 0.0001
Right	**1513** ± 165	**1805** ± 205	p = 0.005
Bilateral	**1553** ± 151	**2000** ± 219	p < 0.0001
**NAc core**			
Left	**1151** ± 90	**2501** ± 539	p < .0001
Right	**1175** ± 104	**1302** ± 290	n.s
Bilateral	**1163** ± 87	**1902** ± 279	p < .0001
**NAc shell**			
Left	**1111** ± 93	**2265** ± 581	p < .0001
Right	**1110** ± 160	**1209** ± 162	n.s
Bilateral	**1111** ± 120	**1737** ± 312	p < 0.0001
**IL**			
Left	**685** ± 214	**980** ± 240	p = 0.009
Right	**692** ± 244	**743** ± 267	n.s
Bilateral	**689** ± 228	**861** ± 242	n.s
**PL**			
Left	**614** ± 206	**854** ± 121	p = 0.003
Right	**615** ± 202	**606** ± 167	n.s
Bilateral	**614** ± 203	**730** ± 130	n.s

Data are shown as means ± standard deviations for each hemisphere. Significances of the ‘Group’ effect are listed in the right-hand column. *BLA* basolateral amygdala, *NAc* nucleus accumbens, *IL* infralimbic cortex, *PL* prelimbic cortex, *n.s.* not significant (p > 0.05).

In the BLA, a 2-way RM-ANOVA revealed significant main effects of both ‘Group’ (*F*_(1,23)_ = 33.14; p < 0.0001) and ‘Hemisphere’ (*F*_(1,23)_ = 23.27; p < 0.0001). We found a significant interaction between both factors (*F*_(1,23)_ = 10.02; p = 0.004). Post-hoc analysis showed a strong c-Fos increase in the left hemisphere of STIM animals, compared to its right counterpart (p < 0.0001) and to c-Fos levels in the left BLA of SHAM animals (p < 0.0001). In addition, c-Fos levels were significantly elevated in the right BLA of STIM versus SHAM animals (p = 0.005).

In the NAc core, main effects of ‘Group’ (*F*_(1,22)_ = 64.72; p < 0.0001) and ‘Hemisphere’ (*F*_(1,22)_ = 30.78; p < 0.0001) were found. The interaction between both factors was also significant (*F*_(1,22)_ = 33.41; p < 0.0001). Post-hoc analysis showed increased c-Fos expression in the left NAc core of STIM animals, compared to its right counterpart (p < 0.0001) and to the left hemisphere of SHAM animals (p < 0.0001).

In the NAc shell, both ‘Group’ (*F*_(1,23)_ = 39.52; p < 0.0001) and ‘Hemisphere’ (*F*_(1,23)_ = 35.16; p < 0.0001) showed significant differences. In addition, there was a significant interaction between both factors (*F*_(1,23)_ = 35.07; p < 0.0001). Post-hoc analysis showed increased c-Fos expression in the left NAc shell of STIM animals, compared to the right hemisphere in STIM animals (p < 0.0001) and to the left NAc in SHAM animals (p < 0.0001).

In the IL, both ‘Group’ and ‘Hemisphere’ showed significant main effects (*F*_(1,23)_ = 3.29; p < 0.0001 and *F*_(1,23)_ = 22.73; p < 0.0001, respectively). In addition, there was a significant interaction between both (F_(1,23)_ = 25.78; p < 0.0001). Post-hoc analysis showed increased c-Fos expression in the left IL of STIM animals, compared to the right hemisphere in STIM animals (p < 0.0001) and to the left IL in SHAM animals (p = 0.009).

In the PL, significant effects were found for ‘Hemisphere’ (*F*_(1,23)_ = 34.86; p < 0.0001) and the interaction between ‘Group’ and ‘Hemisphere’ (*F*_(1,23)_ = 34.96, p < 0.0001). Post-hoc analysis showed increased c-Fos expression in the left PL of STIM animals, compared to the right hemisphere in STIM animals (p < 0.0001) and to the left PL in SHAM animals (p = 0.003).

### Widespread high c-Fos expression in the left hemisphere of STIM rats

**Figure 6:**
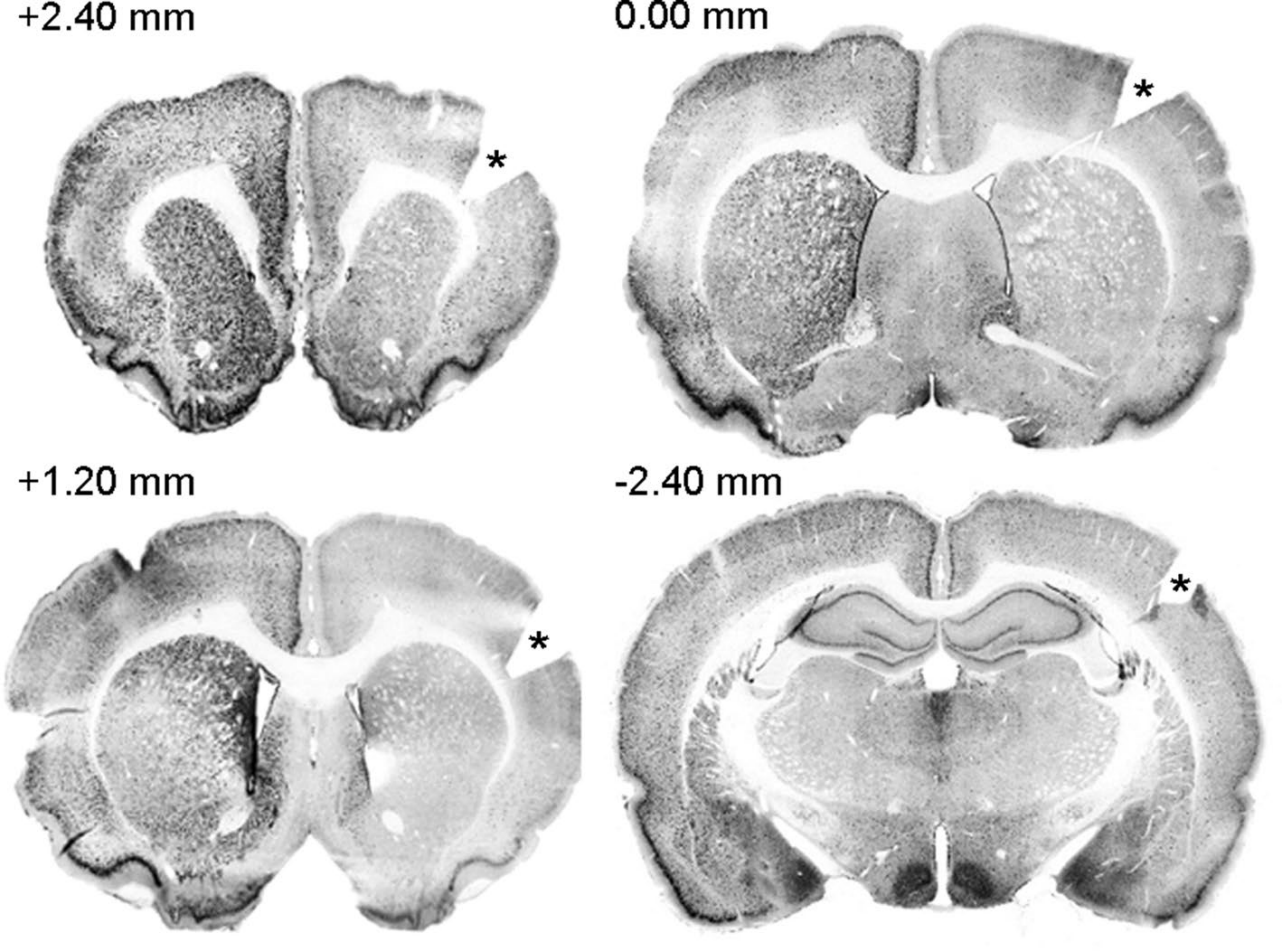
Left hemisphere-wide elevated c-Fos expression at different positions with respect to bregma. One representative animal is shown, but all STIM rats had a similar general pattern. Blackness represents c-Fos expression. Right hemispheres are depicted on the right side and marked by a slit (*) in the cortex.

Given the remarkably high c-Fos intensity in the left-sided ROIs of animals receiving bilateral BST stimulation, we wondered whether the observed c-Fos increases were specific to these ROIs, or rather extended to a larger region in the left hemisphere. To evaluate this, we imaged full coronal sections, anterior and posterior to the electrode, using a fluorescent microscope with 5X magnification (see Supplement for details). Figure 6 illustrates that there was indeed a widespread and unanticipated lateralization, particularly in the more anterior sections, with higher c-Fos intensities in the left hemisphere of bilaterally stimulated rats.

**Figure 7:**
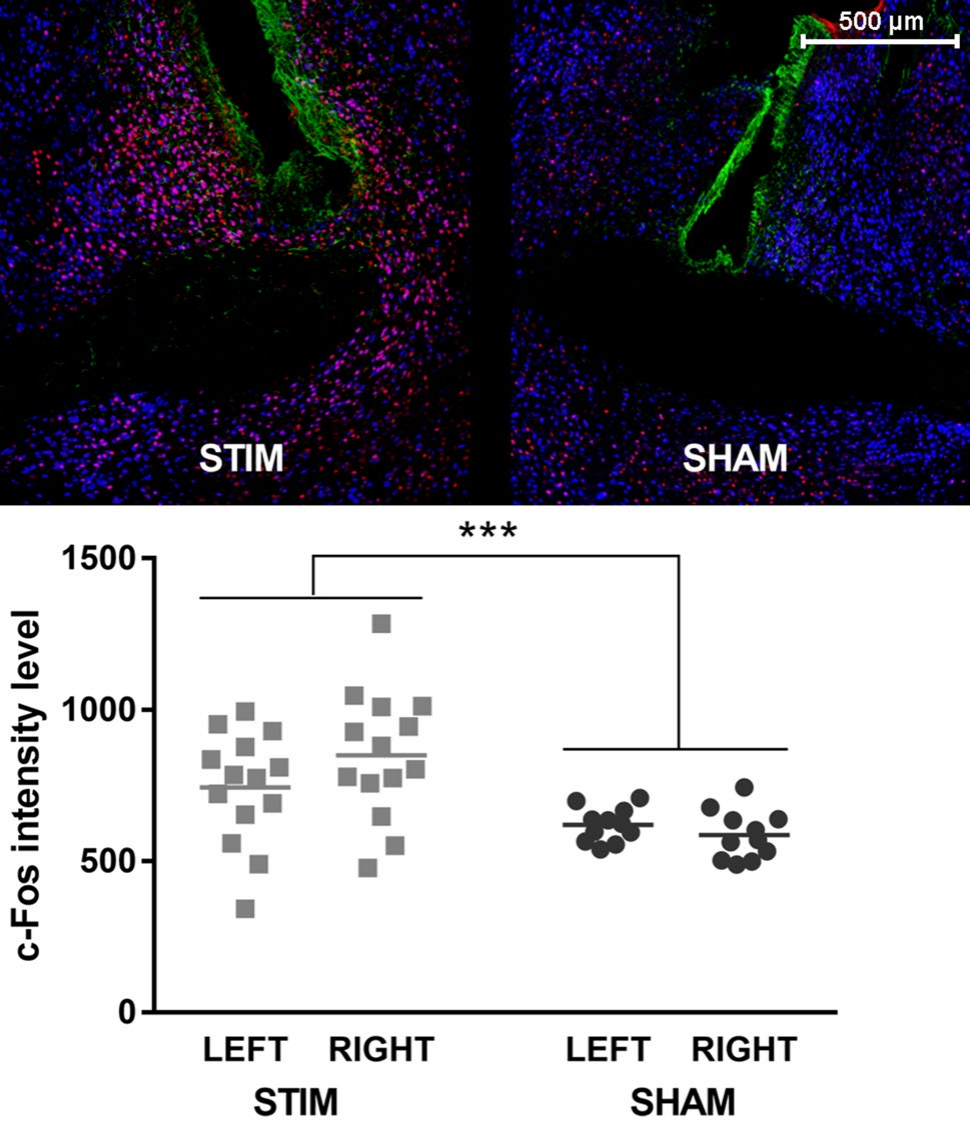
Neuronal c-Fos expression around the electrode tips. Representative images of immunostained cross-sections at the level of the electrode tips in a STIM (left upper panel) and a SHAM animal (right upper panel). Green staining represents GFAP and gliosis surrounding the electrode tip, blue cells are neurons (NeuN), and c-Fos expression is depicted in red. The bottom panel shows average neuronal c-Fos intensity in a 500-µm radius around the electrode tip for all STIM (n = 14, squares) and SHAM (n = 11, circles) animals. Individual data and means are shown, ***p < 0.001.

It is highly unlikely that this left-sided lateralization was the result of unintentional differences in the stimulation that was applied to both hemispheres. We have no evidence for equipment malfunctioning, and the behavioral results were very comparable to those that we obtained previously with a different stimulator^19^. To further examine the possibility of any involuntary differences in stimulation (for example, more stimulation in the left hemisphere and/or no stimulation in the right hemisphere), we analyzed neuronal c-Fos expression in the immediate vicinity (500-µm radius) of the electrode tip (see Supplement for details) (Fig. 7, upper panels). In STIM animals, we expected the neurons around the stimulation site in both hemispheres to stain positive for c-Fos due to direct (i.e., non-trans-synaptic) stimulation effects^55^. Note that this was an exploratory analysis for which the experiment was not specifically designed, and at the level of the BST, only sections at bregma were collected and stained. However, this was not necessarily the position of the center of the electrode tip. Sometimes the bregma section contained only the border of the electrode or was even just adjacent to it. Despite this variability, the analyzed 500-µm radius should give a good indication of the average c-Fos intensities around the electrode tip. Statistical analyses indicated that c-Fos intensities were indeed higher in stimulated than non-stimulated animals, with no differences between hemispheres (2-way RM-ANOVA, effect of ‘Group’ (*F*_(1,23)_ = 16.35; p < 0.001), no effect of ‘Hemisphere’ (*F*_(1,23)_ = 0.77; p = 0.39) and no interaction (*F*_(1,23)_ = 2.87; p = 0.10)), suggesting that STIM rats received balanced bilateral stimulation, as intended (Fig. 7, bottom panel).

## Discussion

In this study, we evaluated neuronal c-Fos expression in anxious, context-conditioned rats (Experiment 1) and in conditioned animals receiving anxiolytic, bilateral high-frequency electrical stimulation in the BST (Experiment 2). Particularly, we aimed to investigate potential asymmetries in c-Fos profiling between left and right hemispheres in several regions of interest within the anxiety network.

In Experiment 1, both behavioral indices of anxiety, freezing and startle potentiation, were significantly increased in ANX versus CTRL animals, indicating successful conditioning to the context. We found that the medial (STMA), but not lateral (STL), subdivision of the anterior BST displayed increased c-Fos expression in ANX versus CTRL rats (Cohen’s *d*_s_: 1.53). The primary involvement of the STMA is in line with our prior research, including rodent PET imaging^14^ and subsequent intervention studies^15,19^. Said imaging study also indicated a stronger hypermetabolism in the left than right BST, although this was not formally tested. Additional evidence for the importance of the STMA for anxiety and stress responses comes from studies showing that electrical activation of the STMA, but not the STL, results in increased corticosterone levels and blood pressure^56,57^, and the observation that STMA neurons are predominantly active during contextual freezing, whereas STL neurons display increased firing during non-freezing episodes in the same animals^58^. Our data corroborate the STMA’s role in anxiety, but moreover suggest an asymmetry in STMA functioning, since c-Fos levels were only significantly elevated in the left, but not right STMA in ANX vs CTRL rats. To the best of our knowledge, this is the first report of BST lateralization in anxiety responses. Furthermore, in the BLA, we found that non-anxious CTRL animals displayed an asymmetric activity pattern (stronger c-Fos intensities in the right than left BLA), whereas ANX animals did not. The right-left asymmetry observed within the CTRL group was rather strong (Cohen’s *d*_z_: 2.16), but with only 6 animals per group, the between-group difference in the right hemisphere was not significant, although numerically, CTRL rats had higher c-Fos levels than ANX rats (Cohen’s *d*_s_: 1.09). As mentioned above, several studies have reported BLA lateralization in contextual fear^10,29–34^, typically indicating stronger involvement of the right BLA in processing of threat cues. Combined with our findings in the BST, these data suggest some cross-hemispheric correlation between the left STMA and the right BLA during expression of anxiety. Further research is necessary to investigate the interactions between these structures, preferably using techniques with a high temporal resolution, given the distinct temporal activation patterns of the BLA and BST during expression of anxiety^7,10,59,60^. Finally, we evaluated neuronal activation in the NAc, core and shell, but found no group differences. Previous studies have investigated the NAc in contextual and cued fear conditioning, but have not been able to reach a consensus on its specific involvement, although it has been implicated in avoidance of threatening situations^45,47,61–67^. Our data suggest that the neuronal cell bodies in this structure (the technique is unable to pronounce upon a potential role of passing fibers^68^) do not mediate contextual fear responses in this particular behavioral procedure.

In Experiment 2, context-conditioned animals received either electrical BST (STIM) or no (SHAM) stimulation. STIM animals showed significantly lower freezing and startle responses than SHAM rats, replicating the anxiolytic effects of bilateral BST stimulation^19^. As mentioned above, the exact mechanism of action of DBS in the BST is yet to be elucidated, but it is noteworthy that the anxiolytic effects of benzodiazepines have been suggested to (partially) depend on GABA receptor activation in the BST^12,69,70^. This seems in line with the hypothesis that DBS may trigger a local release of GABA^22^. Note, however, that the BST is a complex structure with efferent as well as intrinsic GABAergic connections, greatly complicating definitive conclusions in this regard and requiring further research. Following the behavioral test, we evaluated c-Fos expression in the BLA, NAc, IL and PL. Based on the findings in Experiment 1, we hypothesized that BST stimulation might restore the asymmetric c-Fos profile in the BLA that we had observed in non-anxious animals. We did not expect group differences for the NAc, although direct (i.e., non-transsynaptic) c-Fos activation of this region (located 1.20 mm anterior to the electrode), was possible due to the monopolar electric field which has an approximate 1-mm spread^71^. In addition, we examined neuronal activity in IL and PL, which have been implicated in down- and upregulation of fear responses, respectively^65,72,73^. Accordingly, we assumed that STIM animals might show stronger activation of IL and weaker activation of PL than SHAM animals. In line with other studies^74–76^, we observed behavioral differences between non-operated rats (in Experiment 1) and those that had undergone intracranial implantations (in Experiment 2). Specifically, visual comparison of Figs. 2 and 5 suggests that context-conditioned animals in Experiment 1 show lower levels of freezing and startle potentiation. Whether this represents differences in the propensity to freeze or startle, or genuine differences in overall fear or stress levels remains speculative, but it should be kept in mind that such variations may also reflect on the c-Fos expression levels.

In line with the findings of Experiment 1, we observed higher c-Fos intensities in the right BLA of STIM (non-anxious) versus SHAM (anxious) animals (Cohen’s *d*_s_: 1.55). This increase was, however, insufficient to achieve the expected asymmetric profile (right > left BLA, in STIM animals), given a surprising and marked increase of c-Fos expression throughout much of the left hemisphere (Cohen’s *d*_z_ for the comparison between both hemispheres of STIM animals ranging from 1.28 to 1.85 for the different ROIs, and Cohen’s *d*_s_ for the comparison of the left hemisphere between both groups ranging from 1.29 to 3.23). This pervasive unilateral c-Fos elevation hampered the interpretation of the abovementioned hypotheses for each ROI, but is noteworthy in its own right.

Exploratory analyses (see also Supplement) suggested a widespread increase in c-Fos expression in the left hemisphere of STIM animals, surpassing the predetermined ROIs, and more pronounced in regions anterior to the stimulation target than in posterior areas. In addition, local c-Fos expression surrounding the electrode tips did suggest a comparable delivery of current at the stimulation site, as intended with the bilaterally identical stimulation settings that were used. Note that, even though seizures following electrical BST stimulation have been reported^20,77^, we did not observe any signs of this in our animals, largely refuting the idea that the strong widespread c-Fos increase would be due to such seizures. A possible explanation for the unilateral spread of c-Fos throughout the left hemisphere lies in the structural connectivity properties between the BST and other parts of the limbic system. According to Avery et al., the BST has stronger structural connections to parts of the left limbic system, including the left caudate, left amygdala and left NAc^8^. In line with this, anatomical tracer studies have shown predominantly ipsilateral connections between the BST and other gray matter regions^78–80^. The unilateral increase in c-Fos expression could rely on these strong inter-limbic connections in the left hemisphere^8^, altered functional connectivity in a state of anxiety (with stronger activation of the left STMA), or an interaction between both.

Note that the c-Fos technique has limitations: it offers a one-time snapshot of activated neurons, cannot discriminate between different cell types (e.g., inhibitory versus exhibitory), does not provide information about further trans-synaptic processing, and cannot distinguish between direct stimulation effects and actual neuronal activity^55^. For example, although neurons around the electrode tip stain positive for c-Fos as a consequence of electrical stimulation, this does not imply functional activity in this region. Additionally, it is plausible that electric current was transferred directly through fiber bundles (which may be structurally different in both hemispheres, as mentioned above) such as the anterior tract (which connects the BST to cortical structures via the NAc and caudate^68^), thereby increasing c-Fos levels in distant connections without necessarily causing a functional activation in these cells. Regardless, the asymmetric c-Fos expression pattern following bilateral BST stimulation remains remarkable.

In summary, the present study provides evidence for lateralized c-Fos expression in the limbic system during the expression of contextual fear and anxiolytic high-frequency electrical stimulation of the BST in rats, particularly in the medial anterior BST and in the BLA. Further research is needed to unravel the neurobiological underpinnings of the extensive left-sided c-Fos spread following bilateral stimulation and its relevance toward potential unilateral manipulations of the limbic system (e.g., unilateral DBS).

## Supplementary information

Supplementary Information.

## Data Availability

Data can be found here (https://osf.io/uhxwg).
