# Supplementary material for: c-Fos expression following context conditioning and deep brain stimulation in the bed nucleus of the stria terminalis in rats
Source: Sci Rep. 2020 Nov 25;10:20529. doi: 10.1038/s41598-020-77603-z (PMC7688637; doi:10.1038/s41598-020-77603-z)
Supplement: Supplementary file 1 — Supplementary Information. [file 41598_2020_77603_MOESM1_ESM.pdf]

Supplement

c-Fos expression following context conditioning and deep brain stimulation in the bed nucleus of the stria terminalis in rats

Kelly Luyck, Isabelle Scheyltjens, Bart Nuttin, Lutgarde Arckens, Laura Luyten

Supplementary Methods

Subjects

Rats were housed separately with food and water available ad libitum. A 14/10 h light–dark cycle (lights on at 7:00 am) and a room temperature of ±19°C were maintained throughout the experiment. All experiments were conducted during the light phase (8:30 am – 6:00 pm). In Experiment 2, a plastic cage divider was used to prevent damage to the surgical wound by cage mates, while still allowing for social interaction.

Equipment

Animals were conditioned in a small animal cage (inner dimensions: 9.4 cm height, 8.2 cm width, and 16.5 cm length) with a grid floor, through which foot shocks were delivered. The grid floor consisted of six 5-mm-diameter stainless-steel bars spaced 10 mm apart (Med Associates, Inc. Fairfax, VT, USA). The cage was fixed on a response platform and located inside a ventilated sound-attenuating box (Med Associates). A dim red light was continuously on. The freezing behavior of the animals was recorded by a video camera (DCR-SR55E Super NightShot Plus; Sony) positioned in front of the test cage. In addition, the startle reaction of the rats generated a pressure on the response platform and analog signals were amplified, digitized, and processed by software (Startle Reflex, version 5.95; Med Associates). The presentation and sequencing of the acoustic stimuli and foot shocks were controlled by the same software. One of two loudspeakers, both located 7 cm behind the rat holder, was used to deliver a continuous white background noise (55 dB), the other speaker delivered the startle probes (white noise, 100 dB, 50 ms). The startle response was defined as the first peak accelerometer voltage that occurred during the first 100 ms after onset of the startle probe and was measured on an arbitrary scale ranging from 0 to 2047. The startle platform and loudspeakers were calibrated before each experiment. The cage was cleaned with 70% ethanol in between rats.

Context conditioning procedure

Experiment 1

Habituation. On the first day, rats were placed in the startle box for a total of 20 minutes. During the first 5 min (acclimation phase), only background noise (55 dB) was present. Next, 30 acoustic startle stimuli (100 dB, 50 ms) were administered with a fixed inter-trial interval (ITI) of 30 s. This first session was implemented to habituate the animals to the new context and stabilize startle responses before any experimental manipulations took place. Data from this session were not analyzed.

Pre-test. On day 2, rats underwent a Pre-test identical to the Habituation session. In this phase, baseline measurements of anxiety were collected, i.e. freezing and startle. Percentage freezing during the 5-min acclimation phase was scored by an observer, blinded to the group division. Startle measurements were

collected automatically from the Startle Reflex software. Based on their Pre-test startle values, rats were matched into two groups with comparable startle responses (ANX, n = 9; CTRL, n = 7).

Training. On day 3, after the 5-min acclimation phase, rats received 10 electrical foot shocks (0.8 mA, 250 ms; or 0 mA in CTRL animals) with a variable ITI of 60 – 180 s. At this stage, the rats were conditioned to the context. The total duration of this session was 30 min.

Post-test. On day 4, the animals were tested using a 20-min protocol identical to that of the Pre-test. During Post-test, ANX animals were expected to express anxiety in the context where they previously received electrical shocks, as quantified by increased freezing during acclimation and startle potentiation.

Experiment 2

The standard conditioning procedure was followed. Following the Pre-test, animals were assigned to two groups, STIM (n = 17) and SHAM (n = 14). All rats received shocks during Training and were therefore conditioned to the context. STIM animals received electrical BST stimulation prior to and during the Post-test, as detailed below.

Electrical stimulation

For STIM animals in Experiment 2, high-frequency electrical stimulation of the bilateral BST was initiated in a home cage 1 h before and continued during the Post-test. We used a fixed frequency of 130 Hz and pulse width of 220 µs, whereas the amplitude was determined for every animal individually. Amplitudes were increased gradually until persistent side effects occurred (such as excessive shaking, urination, muscle spasms). Next, amplitudes were set just below this threshold. Stimulation of the bilateral BST was performed using two separate, isolated channels and identical parameters. We used monopolar stimulation (the reference was one of the fixation screws on the skull) with biphasic, counterbalanced pulses to avoid tissue damage.

Immunohistochemistry

In Experiment 1, six representative rats of both groups were selected to reflect the group average for freezing and startle measurements as accurately as possible (ANX: n = 6, CTRL: n = 6). This sample size is in accordance with what is commonly used in the c-Fos literature (typically n = 4-6 per group). Note that brain tissue sectioning and subsequent c-Fos analysis was only conducted for this subset. For Experiment 2, to increase power, all animals with correct electrode placement were included (STIM: n = 14, SHAM: n = 11).

Tissue preparation. Two hours after initiation of the Post-test, animals were deeply anesthetized by an intraperitoneal injection of pentobarbital (2.5 ml, Nembutal, CEVA Santé Animale, Brussels, Belgium). Next, the animals were perfused with saline and a 4% paraformaldehyde dilution in phosphate-buffered saline (PBS). Brains were removed and post-fixed for 24 h in 4% paraformaldehyde. Subsequently, samples were rinsed in water during 24 h and stored at 4°C in PBS. Fifty-µm thick free-floating serial sections were prepared on a Vibratome (Microm HM 650 V, Thermo Scientific, Walldorf, Germany) and collected in 24-well plates.

Triple staining procedure. In Experiment 1, we evaluated NAc (core and shell), BLA and BST (STMA and lateral anterior division (STL)) activity. One representative cross-section was chosen for each structure based on Paxinos coordinates: +1.20 mm (NAc), 0.00 mm (BST) and -2.40 mm (BLA), with respect to bregma (+ indicates anterior, - posterior). In Experiment 2, analysis of the BST was hampered by the

placement of the electrodes (i.e., gliosis, edema, rupturing of tissue), which was therefore not a primary region of interest. Instead, we evaluated c-Fos expression in the BLA and NAc, as well as in two additional cortical structures: infralimbic and prelimbic cortex (IL and PL, 2.40 mm anterior to bregma, atlas figure is at +2.52 mm). For each structure of interest, within one animal, three consecutive slices were selected that comprised the abovementioned cross-sections. Note that for each structure, all slices were stained simultaneously**.** This approach decreased variability in c-Fos intensities and allowed us to directly compare this marker of neural activity between groups or hemispheres within the same structure.
Sections were rinsed in PBS-T (0.3% Triton) and incubated in normal goat serum (Chemicon International, Temecula, CA) for 1 h (Tris-NaCl blocking buffer (TNB); 1:5). Next, they were incubated overnight with primary antibody diluted in PBS-T (0.5% Triton). Following antibodies were used: polyclonal rabbit anti-cFos, developed and characterized in-house (1:2000) (Van der Gucht et al., 2000), monoclonal mouse anti-NeuN IgG1, clone 60 (MAB377, Millipore, Darmstadt, Germany; 1:300) and polyclonal chicken anti-GFAP (NBP1-05198, Bio-techne Ltd, Abingdon, UK; 1:5000). The next day, sections were rinsed and first incubated with biotinylated polyclonal goat anti-rabbit IgG secondary antibody (E0433, Dako, Glostrup, Denmark; 1:250) for 2 h. After rinsing in PBS-T, the sections were incubated with a secondary antibody mixture complemented with Streptavidine-Cy5 conjugate (A1011, Life Technologies, Ghent, Belgium; 1:250), diluted in TNB for 2 h. Following antibodies were used: AlexaFluor 488 pre-adsorbed polyclonal goat anti-mouse IgG (ab150117, Abcam, Cambridge, UK; 1:250) and Cy3 pre-adsorbed polyclonal goat anti-chicken IgY H&L (ab97145, Abcam, Cambridge, UK; 1:250). Finally, slices were mounted, incubated with DAPI (2 µl/100 ml PBS, 32670, Sigma-Aldrich) for 30 min and coverslipped with Mowiol (4-88 Mowiol, Sigma-Aldrich, St-Louis, MO, USA).

Image acquisition. Projection images of the regions of interest (ROIs) were acquired through an inverted FV1000 confocal microscope (IX81, Olympus; Aartselaar, Belgium) using a 20X objective (NA: 0.75) at a resolution of 512x512 µm. Upper and lower limits of the slice were determined in the Z-plane. Pictures were acquired as three subsequent z-stacks at 3-µm intervals covering the middle section of the slice, resulting in 9-µm reconstructions. For each structure of interest, three pictures were taken for each hemisphere. Image acquisition was done with FluoView10 software (Olympus, Aartselaar, Belgium) and comprised c-Fos, NeuN and GFAP channels at fixed intensity settings. For all c-Fos imaging, identical laser settings were applied. A low laser intensity of 5% was used, thereby minimizing any risk of bleaching during scanning, and an offset of 5% was used to filter out background fluorescence.

Image analysis. Images were imported in ImageJ (Rasband, ImageJ, National Institutes of Health, Bethesda, MD, http://imagej.nih.gov/ij/, 1997–2014) and ROIs were delineated as 200x200 µm squares situated in each structure of interest (***Fig. 1*** in main article). For each ROI, a neuronal cell mask was obtained by auto-thresholding the NeuN image and detecting cells with circularity levels exceeding 20%. These cells were then automatically delineated and transferred to the c-Fos image, where intensity levels were detected and corrected for total neuronal cell size. Intensity levels were expressed on an arbitrary scale from 0 to 4095. For each unilateral structure, intensity levels were averaged over three consecutive slices. Note that we did not use the number of c-Fos positive neurons as a measure of activity, since most neurons expressed c-Fos under control conditions in our first experiment (i.e., in non-conditioned animals). Instead, we measured intensity levels, which provide information on the *degree* of activity rather than the *number* of active cells.

Supplementary c-Fos data

In the main article, we described two experiments in which we evaluated neuronal activity in the limbic system through c-Fos expression on the protein level. In Experiment 1, we measured c-Fos intensities in regions of the anxiety network in animals without implanted electrodes: context-conditioned rats (ANX) and control animals that did not receive any shocks (CTRL). In Experiment 2, both groups were context-conditioned and, during the post-test, received high-frequency electrical stimulation of the BST (STIM) or no stimulation (SHAM). All findings were discussed in the main Results section, but only a selection of graphs was shown for clarity. Here, all remaining bar plots and tables are provided for the sake of completeness.

Experiment 1

Supplementary Fig. 1 shows individual data points for all ROIs which were not elaborated upon in the main article. Mean c-Fos values and standard deviations for each ROI per hemisphere in both groups are listed in *Suppl. Table 1*. Significances of between-group differences are listed in the right-hand column. Within- and between-group differences were only observed in the BLA and STMA, respectively. The shell of the NAc showed a significant effect of 'Hemisphere' ($F(1,10) = 6.77$; $p = 0.03$), with the left side expressing more c-Fos compared to its contralateral counterpart, in both groups. Again, this finding underlines the importance of evaluating neural activity per hemisphere, rather than analyzing only one hemisphere, or pooling information from both sides of the brain.

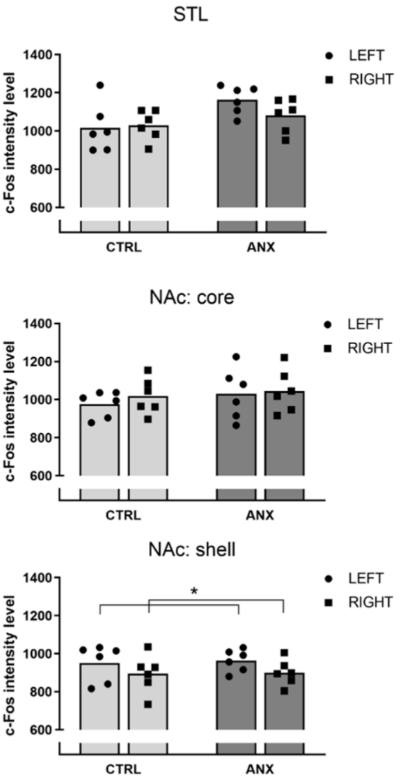

Supplementary Figure 1: c-Fos intensities in Experiment 1. *Neuronal c-Fos expression is shown for the STL (top panel), NAc core (middle panel) and NAc shell (bottom panel). Data are shown as individual data points and means per hemisphere, for CTRL (n = 6) and ANX (n = 6) animals, *p < .05. STL: lateral division of the bed nucleus of the stria terminalis, NAc: nucleus accumbens.*

Experiment 1	CTRL	ANX	Group diff.
STMA			
Left	1149 ± 112	1326 ± 67	p = .004
Right	1187 ± 109	1267 ± 14	n.s.
Bilateral	1168 ± 109	1302 ± 61	p = .03
STL			
Left	1016 ± 128	1164 ± 74	n.s.
Right	1030 ± 78	1081 ± 87	n.s.
Bilateral	1023 ± 97	1123 ± 69	n.s.
BLA			
Left	807 ± 92	788 ± 117	n.s.
Right	925 ± 117	805 ± 103	n.s.
Bilateral	866 ± 102	797 ± 104	n.s.
NAc core			
Left	976 ± 68	1031 ± 134	n.s.
Right	1018 ± 94	1045 ± 113	n.s.
Bilateral	997 ± 76	1038 ± 120	n.s.
NAc shell			
Left	952 ± 97	964 ± 58	n.s.
Right	895 ± 100	900 ± 68	n.s.
Bilateral	923 ± 95	932 ± 37	n.s.

Supplementary Table 1: Overview of neuronal c-Fos intensities in Experiment 1. *Data are shown as means ± standard deviations for each hemisphere. Significances of the 'Group' effect are listed in the right-hand column. STMA: medial anterior division of the bed nucleus of the stria terminalis, STL: lateral division of the bed nucleus of the stria terminalis, BLA: basolateral amygdala, NAc: nucleus accumbens, n.s.: not significant (p > .05).*

Experiment 2

Bar plots with individual data points and means for all ROIs (basolateral amygdala, nucleus accumbens core and shell, infralimbic and prelimbic cortex) are shown in *Suppl. Fig. 2*. As mentioned in the main Results, we observed a pronounced increase in c-Fos intensities in all left-sided ROIs in animals receiving bilateral electrical stimulation of the BST. In addition, the BLA showed a strong increase in c-Fos expression in the right hemisphere in animals receiving BST stimulation versus sham stimulation (Sidak post-hoc test, $t(46) = 3.19$, $p = .005$).

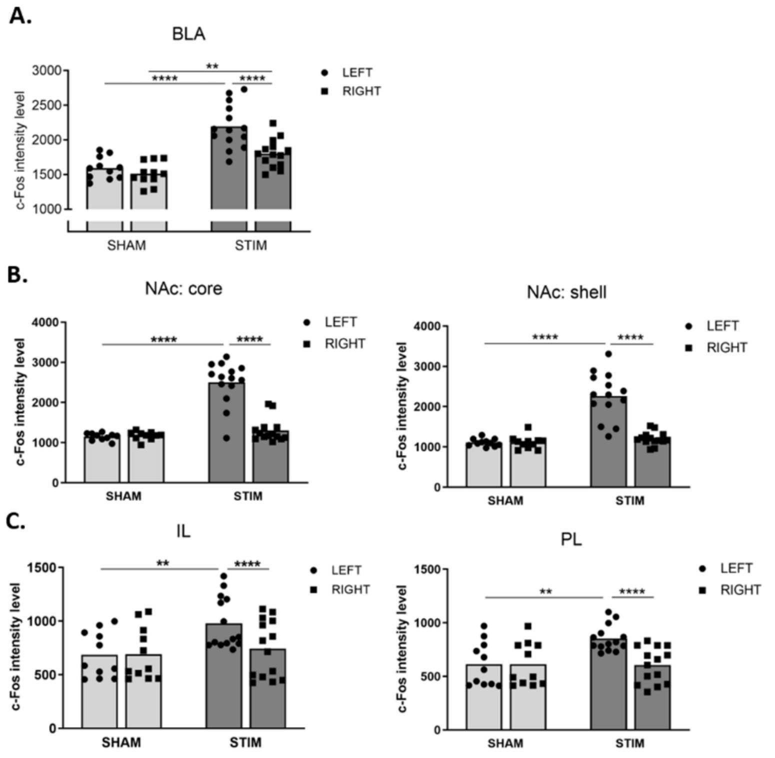

Supplementary Figure 2: c-Fos intensities in Experiment 2. *Neuronal c-Fos expression is shown for the BLA (A), NAc core (B, left panel), NAc shell (B, right panel), IL (C, left panel) and PL (C, right panel). Data are shown as individual data points and means per hemisphere for SHAM (n = 11) and STIM (n = 14) animals, **p < .01, ****p < .0001. BLA: basolateral amygdala, NAc: nucleus accumbens, IL: infralimbic cortex, PL: prelimbic cortex.*

Additional analyses

To gain more insight into the remarkable increase of c-Fos intensity in the left hemisphere accompanying bilateral BST stimulation in Experiment 2, we conducted some exploratory analyses, going beyond the predefined ROIs.

Hemisphere-wide evaluation of c-Fos spread

To assess hemisphere-wide c-Fos expression, we imaged full coronal sections (+2.40, +1.20, 0.00, -2.40 mm with respect to bregma) using a fluorescent microscope with 5X magnification (Zeiss Imager Z1) (*Fig. 6* in main article). Note that these images were not acquired in a confocal manner and c-Fos expression therefore represents the total thickness of the slice (50 µm). In additional contrast to all other analyses, we did not use a neuronal cell mask to quantify c-Fos, given that a neuronal cell count was not possible for this hemisphere-wide analysis, because of extremely high numbers compared to a 200x200 µm ROI analysis. For exploratory quantification of the clearly visible left-right difference, each hemisphere was delineated manually as a ROI in ImageJ, average c-Fos intensities corrected for area size were calculated for each hemisphere, and these were plotted as the ratio of left and right intensities (*Suppl. Fig. 3*). We found that STIM animals had higher lateralization ratios compared to SHAM animals in every coronal section that we investigated. Nominal values were highest in both sections anterior to bregma. None of the 95% confidence intervals of STIM animals comprised the 100 % mark, suggesting lateralization, which was, however, less pronounced in the section posterior to bregma. Finally, SHAM

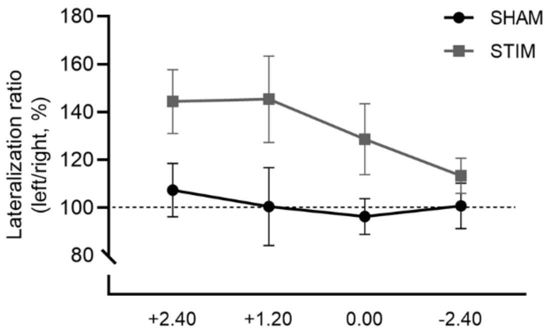

animals showed no hemisphere-wide lateralization and the confidence interval comprised the 100 % mark for every section.

Supplementary Figure 3: Lateralization ratio of hemisphere-wide c-Fos intensities, corrected for area size. *Values above 100% indicate higher c-Fos intensities in the left hemisphere than in the right hemisphere. Data are shown as means and 95% confidence intervals, for both SHAM (n = 11) and STIM (n = 14) animals, at different positions (expressed in mm) relative to the coronal bregma slice (= 0.00 mm).*

Quantification of c-Fos levels adjacent to electrode tips

Next, we investigated in more detail the c-Fos expression in the immediate vicinity of the electrode tips, to verify that left and right BST received comparable electrical stimulation. We expected similar levels of c-Fos in both hemispheres as a result of direct (i.e. non-trans-synaptic) effects of the electrical current. We sectioned the BST of all animals and analyzed cross-sections through (or as close as possible to) the bilateral electrodes (*Suppl. Fig. 4*). Neuronal c-Fos expression in ten 50-µm bins surrounding the electrode was quantified using a custom-made Image J plugin. As described in the main article, there was a clear increase in c-Fos expression around the electrode tip in STIM versus SHAM animals, without significant differences between hemispheres (*Fig. 7* in main article). A more detailed analysis, per 50 µm, did indicate some hemispheric differences in STIM animals, but if anything, c-Fos intensity was slightly *higher* in the right than in the left hemisphere, close to the electrode tip (*Suppl. Fig. 5*). More specifically, the two-way RM-ANOVA in the STIM group indicated no effect of 'Hemisphere' nor 'Distance', but a significant interaction between both ($F(9,117) = 3.89$, $p < .001$), with the difference situated within the 150 µm closest to the electrode (Sidak post-hoc tests on 50-µm bins, $p < .01$). The ANOVA in the SHAM group showed no main effects nor interaction. Note that this fine-grained analysis per 50 µm should be interpreted with sufficient caution because manual delineation of the electrode tip was not always straightforward, as mentioned in the main article. Nonetheless, these exploratory analyses do suggest that STIM rats did not receive less electrical stimulation in the right than in the left BST, in line with the bilateral stimulation protocol that we applied.

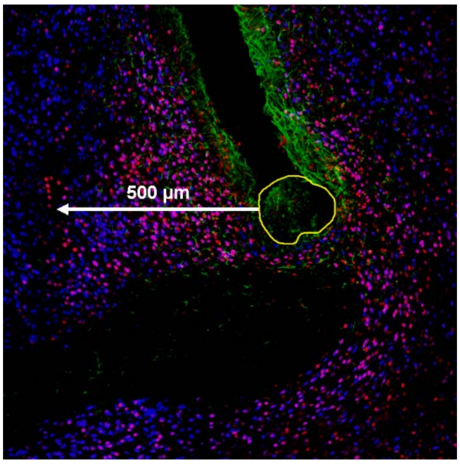

Supplementary Figure 4: Representative image of a cross-section at the electrode tip of a representative STIM animal. *Green staining represents GFAP and gliosis surrounding the electrode tip, blue cells are neurons (NeuN), and c-Fos expression is depicted in red. The yellow line is the manual delineation of the electrode tip from which neuronal c-Fos expression was analyzed in a radius of 500 µm.*

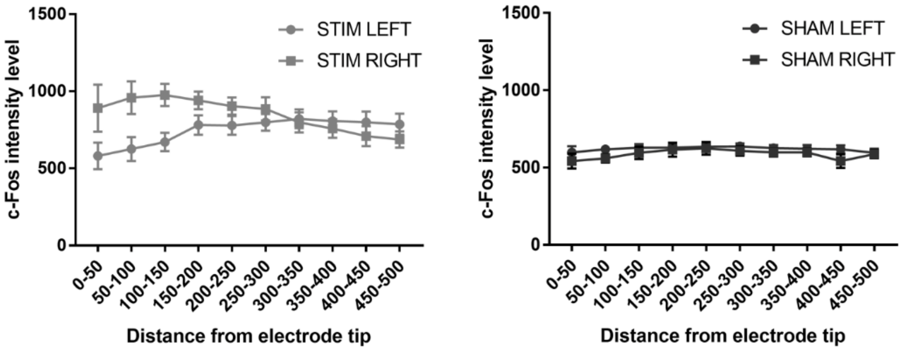

Supplementary Figure 5: Neuronal c-Fos intensities around the electrode tips. *Data are shown as means ± SEM for STIM (n = 14) and SHAM (n = 11) animals. Intensities are expressed on an arbitrary scale between 0 and 4095 for ten bins of 50 µm.*

Representative c-Fos expression in/around the BST in both experiments

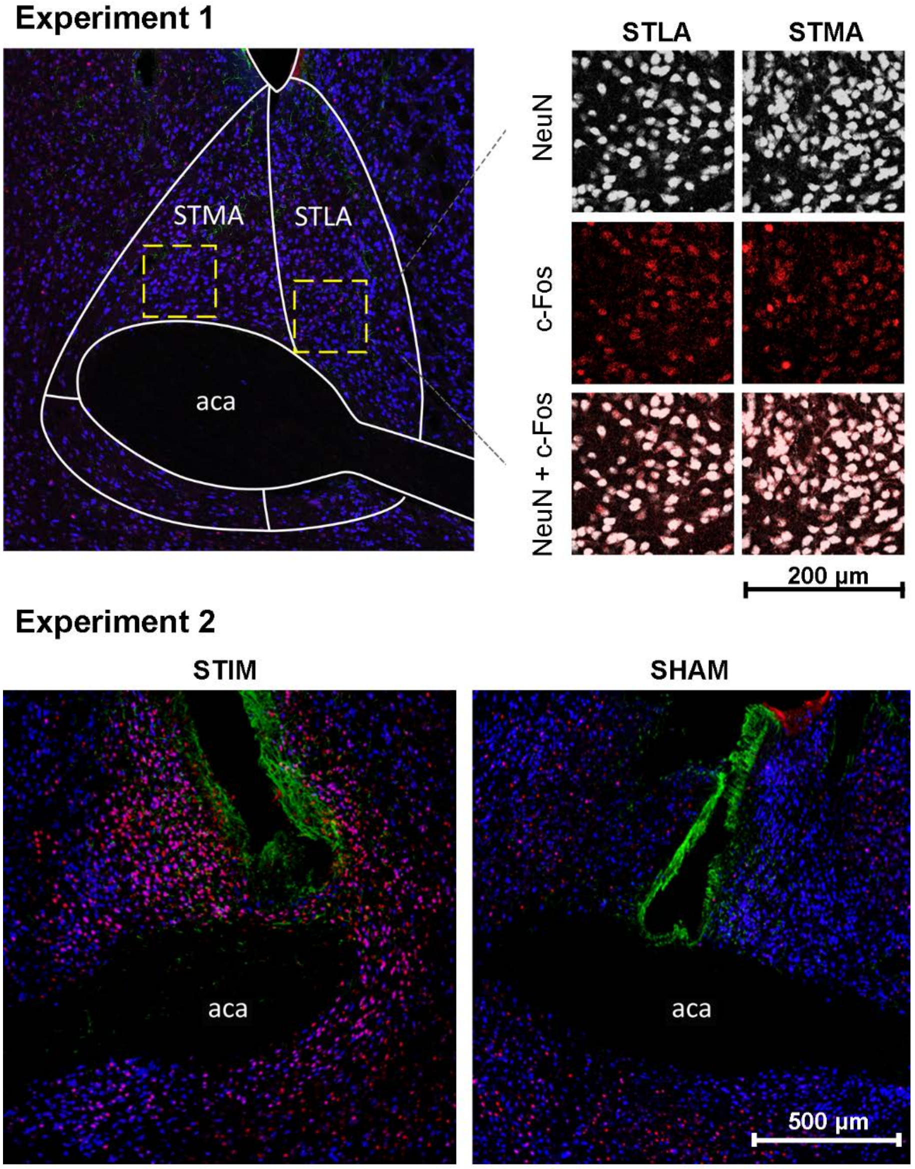

Supplementary Figure 6: Representative immunohistochemistry images at the level of the BST. *The upper panel shows immunostained cross-sections of Experiment 1, the lower panel of Experiment 2. For Experiment 1, an overview image is displayed on the left side and the detailed 200 x 200 µm ROIs from a representative CTRL animal on the right side. Images from ANX animals look similar, as group differences are not visible to the naked eye, but only emerge in the c-Fos intensity analyses. The detailed ROIs show NeuN in white/gray and c-Fos in red. The colocation image shows that c-Fos is primarily expressed in neurons. In Experiment 2, the BST was not analyzed as a ROI per se (given the expected damage due to placement of the electrode tip), but these figures illustrate the typical image that was seen in stimulated (STIM) versus sham (SHAM) animals. Green staining represents GFAP and gliosis surrounding the electrode tip (in Experiment 2), blue cells are neurons (NeuN), and c-Fos expression is depicted in red. aca: anterior part of the anterior commissure, STLA: medial lateral part of the bed nucleus of the stria terminals, STMA: medial anterior part of the bed nucleus of the stria terminalis.*
